# Comparative outcomes of anticoagulant versus antiplatelet therapy in coronary endarterectomy: a meta-analysis

**DOI:** 10.1007/s12055-026-02221-9

**Published:** 2026-06-16

**Authors:** Giuseppe Comentale, Armia Ahmadi-Hadad, Rachele Manzo, Valentina Parisi, Anna Milione, Anna Damiano, Concetta Calanni Macchio, Emanuele Pilato

**Affiliations:** University of Napoli “Federico II” - Via Sergio Pansini N°5, 80131 Naples, Italy

**Keywords:** Coronary endarterectomy, CABG, Anticoagulation therapy, Antiplatelet therapy, Anticoagulant, Warfarin, Aspirin

## Abstract

**Background:**

Coronary endarterectomy (CE) has recently returned to the limelight due to its advantages in achieving full revascularization in very complex cases. However, to date, there are no guidelines concerning the use of postoperative antiplatelet and anticoagulant therapy (ACT).

**Aim:**

In light of this, the present meta-analysis aimed to compare the impact of ACT versus antiplatelet therapy on short- and long-term outcomes after CE.

**Methods:**

PubMed, Embase, and Medline databases were searched without any restriction on the year or the language. Two reviewers screened the studies in two rounds in a blinded manner and extracted the data into an Excel spreadsheet. The third reviewer reconciled discrepancies.

**Results:**

Nineteen studies made our final selection. Short- and long-term mortality was similar between patients treated with ACT and those receiving double antiplatelet therapy (DAPT) (*p* = 0.15 and *p* = 0.98, respectively). Short-term myocardial infarction (MI) was significantly lower among DAPT patients (*p* < 0.001); while during the long-term follow-up, MI events were similar (*p* = 0.77). Dual antiplatelet agents were also compared, suggesting that DAPT with aspirin + clopidogrel and aspirin + ticagrelor regimens lead to similar long-term graft patency (*p* = 0.09).

**Conclusion:**

Postoperative mortality seems to be not affected by the anticoagulant/antiplatelet therapy after CE. DAPT leads to a greater reduction in short-term MI rates; thus, the postoperative use of DAPT seems to offer more benefits compared to ACT therapy. Long-term MI events, however, were comparable. Long-term graft patency analysis of DAPT with aspirin + clopidogrel and aspirin + ticagrelor has not revealed any superiority.

**Graphical abstract:**

**Figure:**
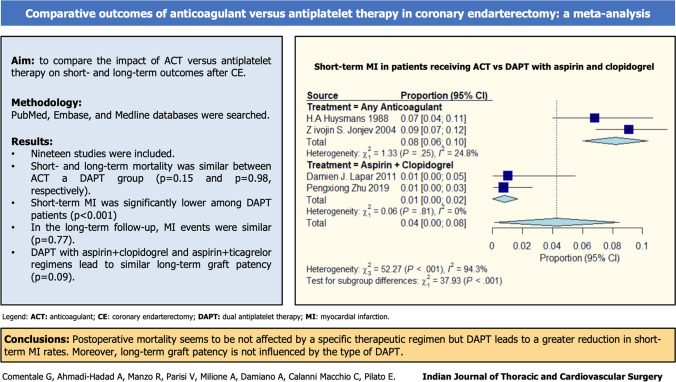

**Supplementary Information:**

The online version contains supplementary material available at 10.1007/s12055-026-02221-9.

## Introduction

Since the 1950 s, coronary endarterectomy (CE) has been developed to treat coronary artery disease [1, 2]. Early investigations, however, found that CE was associated with high postoperative mortality and increased rates of complications such as myocardial infarction (MI) [3, 4]. Many authors suggested that the obstruction of the many smaller branches that really supply the myocardium would have been probably directly involved: the removal of the intima and media exposes an inner very thrombogenic tissue that, in the absence of appropriate therapy, could increase the risk of coronary occlusion or microvascular embolization [4–6]. This probably could explain why CE in addition to coronary artery bypass graft (CABG) is associated with higher mortality and postoperative MI compared to CABG alone in the early postoperative period [7].

As a result, when CABG was developed, CE was quickly forsaken and cardiac surgeons became extremely cautious, considering it only as “last resort” when other approaches proved to be not useful [7–9]. In the case of very severe coronary disease, the distal anastomosis can be a very hard challenge and the target vessel may even be ungraftable. The same problem arises when CABG has to be performed in patients with prior full-metal jacket stenting [10].

For these reasons, CE has recently returned to the limelight due to its advantages for accomplishing full revascularization during CABG [8, 9]. The Society of Thoracic Surgeons (STS) recently published the results about the widest ever reported series of CE; Kelly et al. demonstrated that, even if CE + CABG showed increased mortality and postoperative MIs compared to CABG alone, the risk of concomitant CE is acceptable for those patients requiring advanced revascularization techniques [7].

One of the most significant challenges pertaining to CE is the lack of official guidelines regarding postoperative anticoagulation/antiplatelet therapy. Achieving optimum levels of postoperative antithrombotic therapy for the patients undergoing CE + CABG could contribute to reducing the most common complication occurring after CE, that of graft occlusion, as well as early and late mortality [7–9, 11–14]. The present meta-analysis aims to investigate patients undergoing CE, with concomitant CABG, who received postoperative anticoagulant or dual antiplatelet therapy, and to evaluate outcomes related to short- and long-term mortality, MI, and graft patency.

## Methods

The meta-analysis protocol was registered in PROSPERO on 05/04/2023 with ID CRD42023411383. The outcomes of the study were postoperative mortality, postoperative MI, and graft patency at short- (up to 30 days after the surgery), middle- (from 1 to 6 months), and long-term (after 6 months) follow-up. The follow-up lengths were arbitrarily chosen according to the analysis of the available data and the lengths mostly used in the included studies. As a note, most of the selected studies did not specify the methods they used to assess graft patency, but others clearly reported that it was done using a follow-up coronary angiography.

We stratified the patient cohort into two distinct groups: the dual antiplatelet therapy (DAPT) group and the anticoagulant group. It is of significance to underscore that, in the majority of instances within the anticoagulant group, patients received a sequential regimen of warfarin followed by DAPT/single antiplatelet therapy (SAPT) with aspirin. However, exceptions to this pattern involved the administration of other anticoagulants and exclusively in one case [15] warfarin therapy without DAPT/SAPT. For the subsequent analysis, patients exclusively receiving DAPT were categorized into two distinct groups: one comprising those administered Aspirin in combination with clopidogrel, and the other consisting of patients who received aspirin in combination with ticagrelor.

### Search strategy

The PubMed, Embase, and Medline (via Ovid) databases were searched on 12 March 2023 without any restriction on the year or the language (SF1). MeSH term field tags were not utilized as they could result in missing articles that existed before the relevant MeSH term was introduced. This problem was solved by manually adding the free text terms found within each MeSH term using a MeSH database search. In addition, significant investigations not specifically centered on antiplatelet/anticoagulant treatment for CE patients found among the references of the included studies, yet containing valuable data for meta-analysis, were included. The supplementary material (ST1) provides a list of all additional papers that underwent screening.

**Fig. 1:**
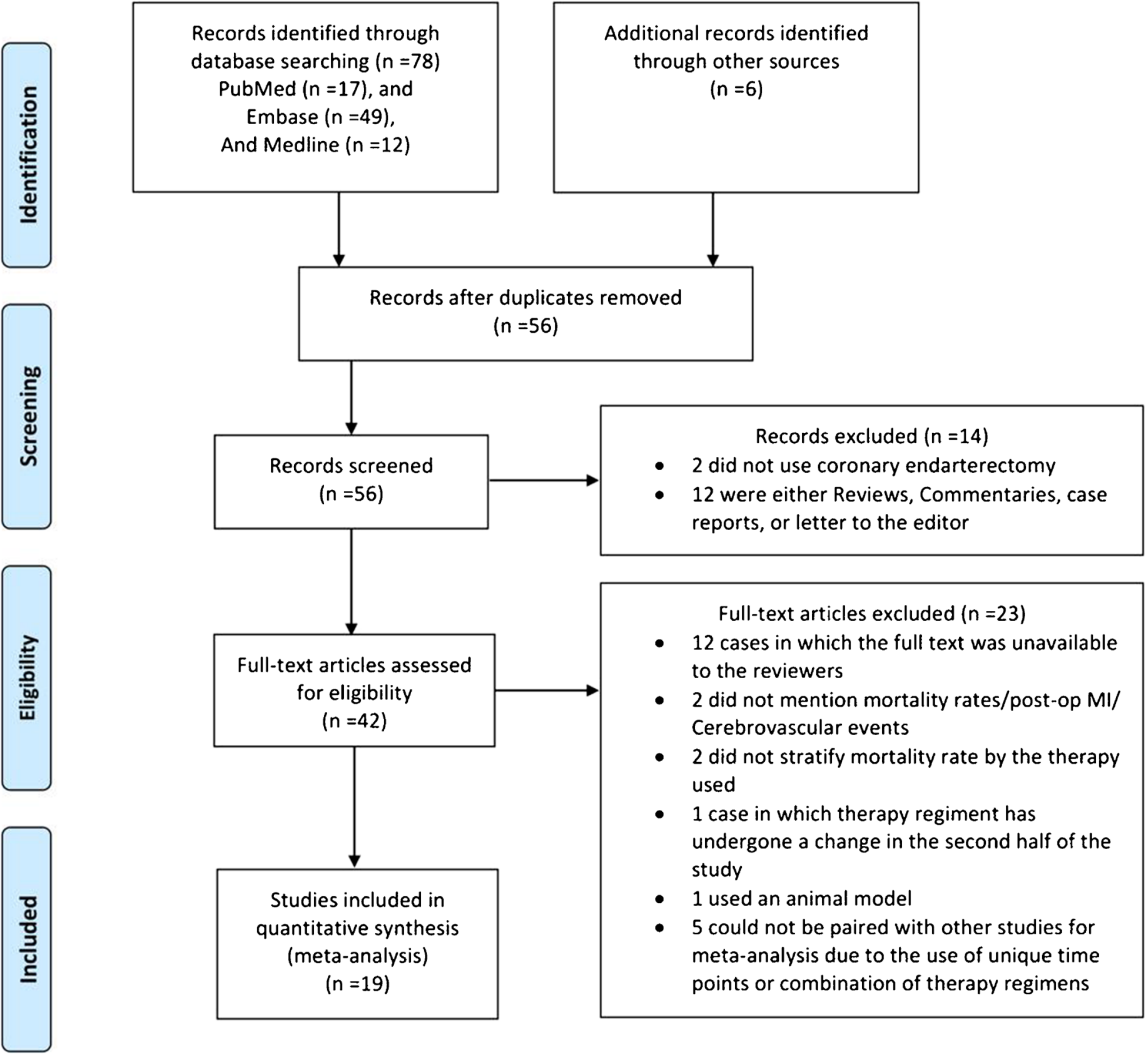
Preferred Reporting Items for Systematic Reviews and Meta-Analysis (PRISMA) flow diagram. Exclusions and inclusions are described in detail

Subsequently, studies were collected in the reference manager software EndNote™. Full search terms are available in the supplementary material. Subsequently, two reviewers (AAH and GC) screened publications in a blinded manner, screening abstracts in the first round and full texts in the second. All discrepancies, where consensus could not be reached through discussion between the two reviewers, were resolved by the third reviewer (EP). Eventually, 19 studies were chosen for meta-analysis. Figure 1 depicts an overview of the screening and selection procedure.

### Inclusion and exclusion criteria

Studies investigating the use of antiplatelet and/or anticoagulant therapy in patients who underwent CE were included regardless of the age of the study population. The studies were eligible if they (1) performed CE; (2) measured postoperative mortality, MI events, and major cerebrovascular events; and (3) provided the study’s full text in English. Publications were excluded if (1) they were crossover studies, case studies, reviews, commentaries, case reports, or letters to the editor; (2) they were animal or in vitro studies; and (3) the anticoagulant and/or antiplatelet drugs were not mentioned. It is of significance to note that we had no constraints with regard to on or off-pump methodology for performing the CABG.

### Assessing risk of bias and publication bias

Potential publication bias was assessed by inspection of a funnel plot for asymmetry (Supplementary Figs. S1 and S2). The risk of bias was examined using the ROBINS-I tool (Risk Of Bias In Non-randomized Studies—of Interventions). This tool assesses risk of bias across seven domains: confounding, selection bias, classification of interventions, deviations from intended interventions, missing data, outcome measurement, and selective reporting. These domains ensure a structured and comprehensive evaluation of non-randomized studies.

### Data extraction and synthesis

Data extraction was performed in an independent manner by two reviewers using texts, tables, figures, and supplementary materials. Data on first author name, year of publication, study design (experimental groups, control groups, number of patients per group), male-to-female ratio, age of the patients, anticoagulation and/or antiplatelet protocol, and primary outcomes (postoperation short-term and/or long-term mortality/post-op MI/post-op cerebrovascular events) were collected (Supplementary material, ST2, ST3, ST4, ST5). Most data were presented in the form of mean ± standard deviation. In two cases, WebPlotDigitizer (available from https://automeris.io/WebPlotDigitizer/) was used in order to extract data from figures. All data were placed in a single Microsoft Excel spreadsheet to streamline data processing. Meta-analysis was performed in line with recommendations of the Cochrane Collaboration and the Preferred Reporting Items for Systematic Reviews and Meta-analyses (PRISMA) guidelines [16].

### Statistical analysis

All statistical analyses were performed using RStudio (version 4.4.1) via “Meta” library. A DerSimonian and Laird (DL) random effect model was utilized. All performed analyses were two-tailed with *p* < 0.05 considered statistically significant. Data heterogeneity was assessed with *I*^2^. *I*^2^ > 50% was considered heterogeneous.

## Results

The study selection process is demonstrated using the PRISMA flow diagram in Fig. 1. Seventy-eight studies were initially collected by performing an advanced search in PubMed, Embase, and Medline. Furthermore, an additional cohort of six studies was incorporated into our study sample through the search of the reference list of the retrieved papers.

Only 19 studies were finally selected for the meta-analysis; the reasons for exclusion are demonstrated in Fig. 1.

These included the studies that used antiplatelet treatment [17–26] and studies that merely used anticoagulation therapy [15, 27–34]. Of the 19 studies that made the final selection, some used both anticoagulant and antiplatelet therapy; such cases were assigned to the anticoagulant group.

Table 1 presents comprehensive information regarding the therapy regimens employed and the specific time points at which outcome measurements were conducted, but it was difficult to reconcile the heterogeneous time points of outcome measures between the studies. For these reasons, as no useful data were found about middle-term follow-up, statistical analysis was restricted only to short- (< 30 days after the operations) and long-term (after 6 months) follow-up. Supplementary Table 6 (ST6) details the study designs of all the included papers.

**Table 1:** General overview of the studies included in the analysis

Study	Patients	Age	Male (%)	Treatment(s)	Time points for outcomes
Abid AR et al. (2009) [17]	50	53.56 ± 10.16	90%	At the interval of 2 hours following surgical intervention, patients were administered a combination of aspirin at a dosage of 75 mg and clopidogrel at a dosage of 150 mg. The regimen of 5,000 IU of unfractionated heparin administered every eight hours was initiated concomitantly with aspirin and clopidogrel. Double antiplatelet therapy was given for only 3 months; nevertheless, the administration of aspirin at a dosage of 75 mg was perpetuated indefinitely.	In hospital mortality
El-Gamel A et al. (2021) [18]	22	64 (60–84)	72.73%	Aspirin and clopidogrel were introduced post-surgically. Clopidogrel was stopped after a year; However, aspirin was continued for a lifetime.	Short-term: 30 d Long-term: 1y
LaPar DJ et al. (2011) [19]	99	62.2 ± 10.4	70.7%	All patients who underwent endarterectomy were administered postoperative treatment consisting of Aspirin and Plavix for a duration of no less than three months.	LT mortality: 36m LT MI: 60 m ST MI: periop.
Li H et al. (2022) [20]	94	62.6 ± 7.9	63.20%	In the postoperative period, heparin was administered 6–8 hours following the surgical procedure if the bleeding rate was equal to or less than 100 ml per hour. From postoperative days 1 to 3, a combination of 100 mg aspirin and low molecular weight heparin was administered. Patients underwent Double antiplatelet therapy consisting of 100 mg aspirin and 90 mg ticagrelor. Following this, administration of 100 mg aspirin was prescribed for lifetime.	1 year
Yan H et al. (2020) (AC group) [21]	67	61.4 ± 8.5	74.6%	After the operation, aspirin at the dose of 100 mg and clopidogrel at the dose of 75 mg were given per day. After one-year post-op, patients switched to aspirin monotherapy.	Short-term after operation
Russo M et al. (2017) (DAT group) [22]	52	66.6 ± 7.8	67%	Patients were given 100 mg aspirin and 75 mg clopidogrel per day post-surgery. Therapy regiment was switched to aspirin monotherapy after one year post-op.	7 years
Zhu P et al. (2019) [23]	Periop: 270 Mid-term: 248	64.98	85.55%	If the bleeding was greater than 100 ml/h, patients received low molecular heparin 6 hours post-surgery in the intensive care unit. For the first year post-op, patients were given aspirin 100 mg and clopidogrel 75 mg daily. The aspirin 100 mg was then maintained indefinitely.	1 year, midterm: 51m
Shehada SE et al. (2022) (DAT group) [24]	174	66.7 ± 9.3 From *N *= 326 In the whole study group	88% From *N *= 326 In the whole study group	Dual antiplatelet therapy (DAPT)	1 year
Chow SC et al. (2022) [25]	81	61.9 ± 9.29	82.7%	The usage of double antiplatelet therapy with aspirin and ticagrelor post-op was 90.1%. If the haemorrhage risk was not high, patients were administered with aspirin 160 mg post-extubation and ticagrelor the next day.	20.2 months
Binsalamah ZM et al. (2014) [26]	58	67.3 ± 10.6	90%	Aspirin 80mg/day was initiated on the first day following the surgical procedure. However, patients who underwent endarterectomy received an additional medication, namely Plavix (Clopidogrel 75 mg daily), alongside aspirin, beginning on the first day post-surgery. This combined medication regimen was continued for a duration of three months with the aim of averting the early activation of the coagulation cascade, a phenomenon previously associated with endarterectomy.	In hospital mortality
Kyuchukov D et al. (2020) [27]	24	59.86 ± 9.88	66.7%	Acenocoumarol and aspirin were administered in combination. Intravenous injection of heparin was initiated six hours post-surgical intervention. Subsequently, after an additional six-hour interval, acenocoumarol at a dosage of 6 mg and aspirin at a dose of 80 mg were administered. The infusion was terminated, when INR levels reached the 2–3 range.	30 days
Huysmans HA et al. (1988) [28]	250	56.8 (34–81)	88%	All patients enrolled in the study were subjected to ongoing anticoagulation therapy using coumarin derivatives.	LT mortality: 5.2y LT MI: 4.4 y ST mortality: in-H
Nishigawa K et al. (2017) [29]	205	66.28	85.85%	When chest drainage receded, low molecular weight heparin (5000 units/day) was injected until warfarin started to have effect. With the start of oral ingestion, low-dose aspirin (100mg/day), clopidogrel (75mg/day), and warfarin were given. Warfarin and clopidogrel were stopped, respectively three months and one year post-op, although aspirin was held continuously.	5.4 years
Nishigawa K et al. (2021) [30]	233	65.9 ± 9.5	85.8%	When chest drainage normalized, continuous intravenous injection of low molecular weight heparin (5000 units/day) was started and maintained until warfarin was effective. With the start of oral ingestion, low-dose aspirin (100 mg/day), clopidogrel (75 mg/day), and warfarin were commenced.	5 years
Nemati MH et al. (2015) [31]	84	61.59 ± 9.27	65.47%	Individuals assigned to the coronary endarterectomy+ group were administered a therapeutic regimen consisting of 80 mg of aspirin per diem for a duration of three months, accompanied by the concomitant administration of warfarin, intended to uphold an INR within the range of 2 to 2.5. Following the initial three-month period, the administration of warfarin was terminated.	in hospital mortality (5.49 ± 1.43 days)
Ranjan R et al. (2017) [15]	1000	61.5 ± 5.5	78.2%	Heparin was administered post-coronary endarterectomy, with oral Warfarin given for the following 3 to 6 months	> 6 months MI: 10.5 ± 2.5 m
Fukui T et al. (2005) [32]	11	64 (43–75)	72.72%	Following the surgical procedure, a regimen of low molecular weight heparin (at a dosage of 5000 units per day) was administered until the anticoagulant effects of warfarin were established.	17.3 ± 12.8 months
Kato Y et al. (2012) [33]	37	61.9 ± 10.4	68%	All subjects were administered anticoagulation therapy following their surgical procedures. The administration of low molecular weight heparin commenced six hours after the patients' admission into the intensive care unit. Subsequently, low-dose aspirin and warfarin were initiated once the patients had initiated oral ingestion (The goal INR for warfarin therapy was set at 2.0.), and the administration of these medications was continued forever for all participants. The intravenous administration of heparin was discontinued once the therapeutic effects of warfarin were achieved.	In hospital mortality
Jonjev ZS et al. (2004) [34]	606	55.8 ± 6.2	84.98%	Heparin was normally started 24 hours post-surgery and replaced with coumadin after thrombin and partial thromboplastin times had returned to normal.	30 days

Age is reported either in mean ± SD or median (range)

*d*, days; *m*, months; *y*, years; *LT*, long-term; *ST*, short-term; *MI*, myocardial infarction; periop, perioperative; *INR*, international normalized ratio

### Risk of bias analysis

Risk of bias was assessed using the ROBINS-I tool. The results of this analysis are detailed in Supplementary Table 7 (ST7). The heterogeneity of the timing of outcome measurements was the main obstacle, owing mainly to the limited number of studies performed on CE. We endeavored to address this matter by aggregating studies with proximate time points for outcome assessments. Another constraint that we encountered pertained to the therapeutic regimen used. In most of the studies in which patients were treated with warfarin, the therapy was switched after 3–6 months to various treatments such as DAPT with aspirin and clopidogrel/ticagrelor, or SAPT with aspirin. Due to the restricted number of research done on CE, we could not sub-stratify our analyses further to address this heterogeneity. Ultimately, inspection of the funnel plots suggested the presence of potential publication bias with regard to the outcome of short-term mortality, short- and long-term MI, and graft patency.

#### Short-term mortality in anticoagulant vs aspirin + clopidogrel

**Fig. 2:**
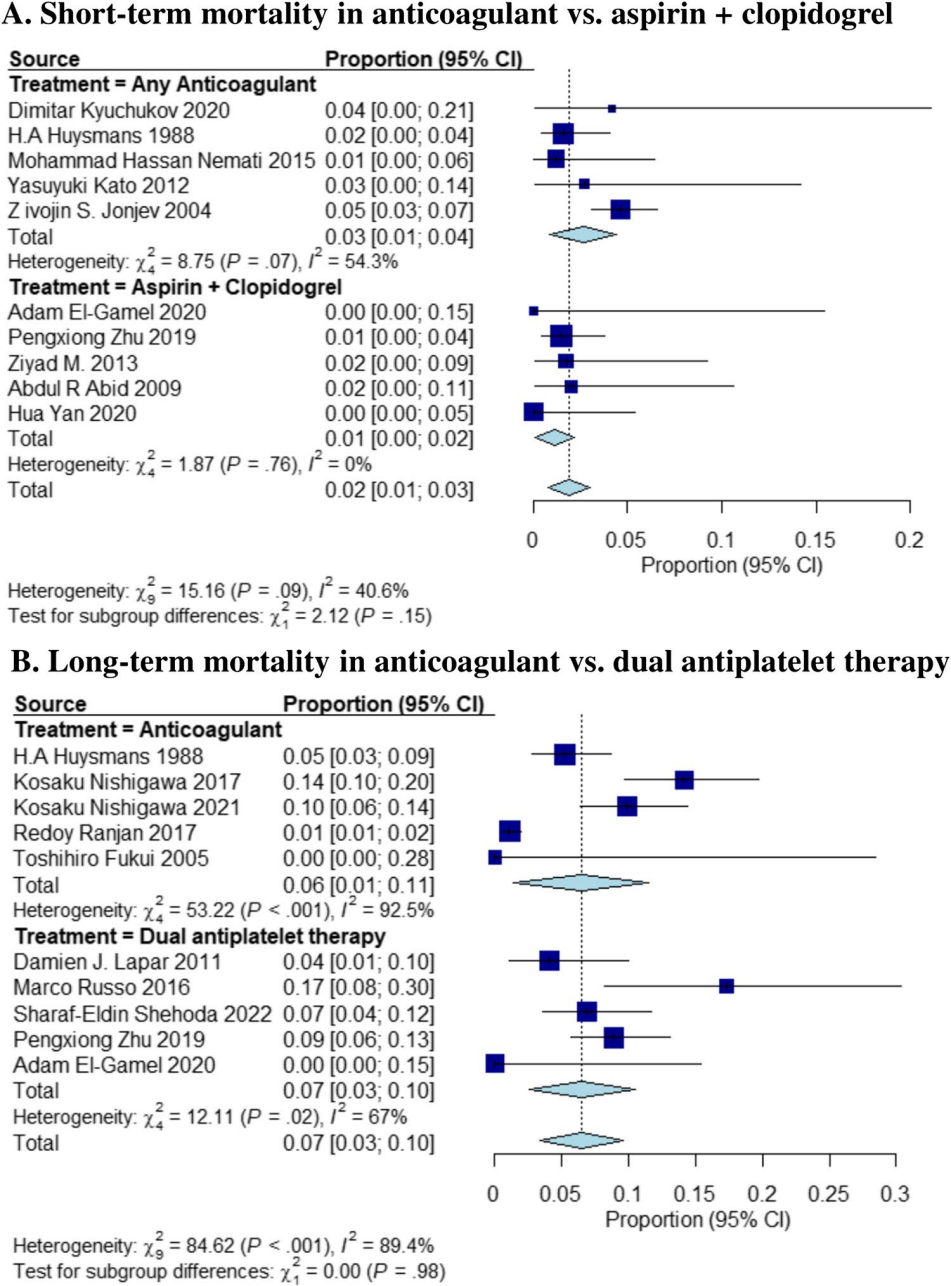
Forest plots showing (**A**) short-term and (**B**) long-term mortality in patients receiving anticoagulants versus those on double antiplatelet treatment (DAPT) with aspirin and clopidogrel

Ten studies measured mortality at time intervals ≤ 30 days after CE in patients treated with either anticoagulant or DAPT with aspirin and clopidogrel [17, 18, 21, 23, 26, 28, 29, 31, 33, 34]. A total of 1468 patients were involved in this assay. A pooled mortality of 3% (95%, 1–4%; *I*^2^ = 54.3%) and 1% (95% confidence interval (CI), 0–2%; *I*^2^ = 0%) was found for anticoagulant and DAPT cohorts respectively. The overall proportion of patients for this analysis was 2% (95% CI, 1–3%; *I*^2^ = 40.6%). No significant difference in short-term mortality was observed between the anticoagulant cohort and the DAPT group (*p* = 0.15) (Fig. 2A). The time intervals used in each study are demonstrated in Table 1.

#### Long-term mortality in anticoagulant vs DAPT

Ten studies measured mortality at an estimated weighted average of 2.8 years (ranging from 1 to 7 years) after CE in patients treated with either anticoagulant or DAPT [15, 18, 19, 22–24, 28–30, 32]. A total of 2294 patients were involved in this assay. A pooled mortality of 6% (95% CI, 1–11%; *I*^2^ = 92.5%) and 7% (95% CI, 3–10%; *I*^2^ = 67%) was found for anticoagulant and DAPT cohorts respectively. The overall proportion of patients for this analysis was 7% (95% CI, 3–10%; *I*^2^ = 89.4%). No significant difference in long-term mortality was observed between the anticoagulant cohort and the DAPT group (*p* = 0.98) (Fig. 2B). Therapy regimens employed in each study are enumerated in Table 1.

#### Short-term MI in anticoagulant vs aspirin + clopidogrel

**Fig. 3:**
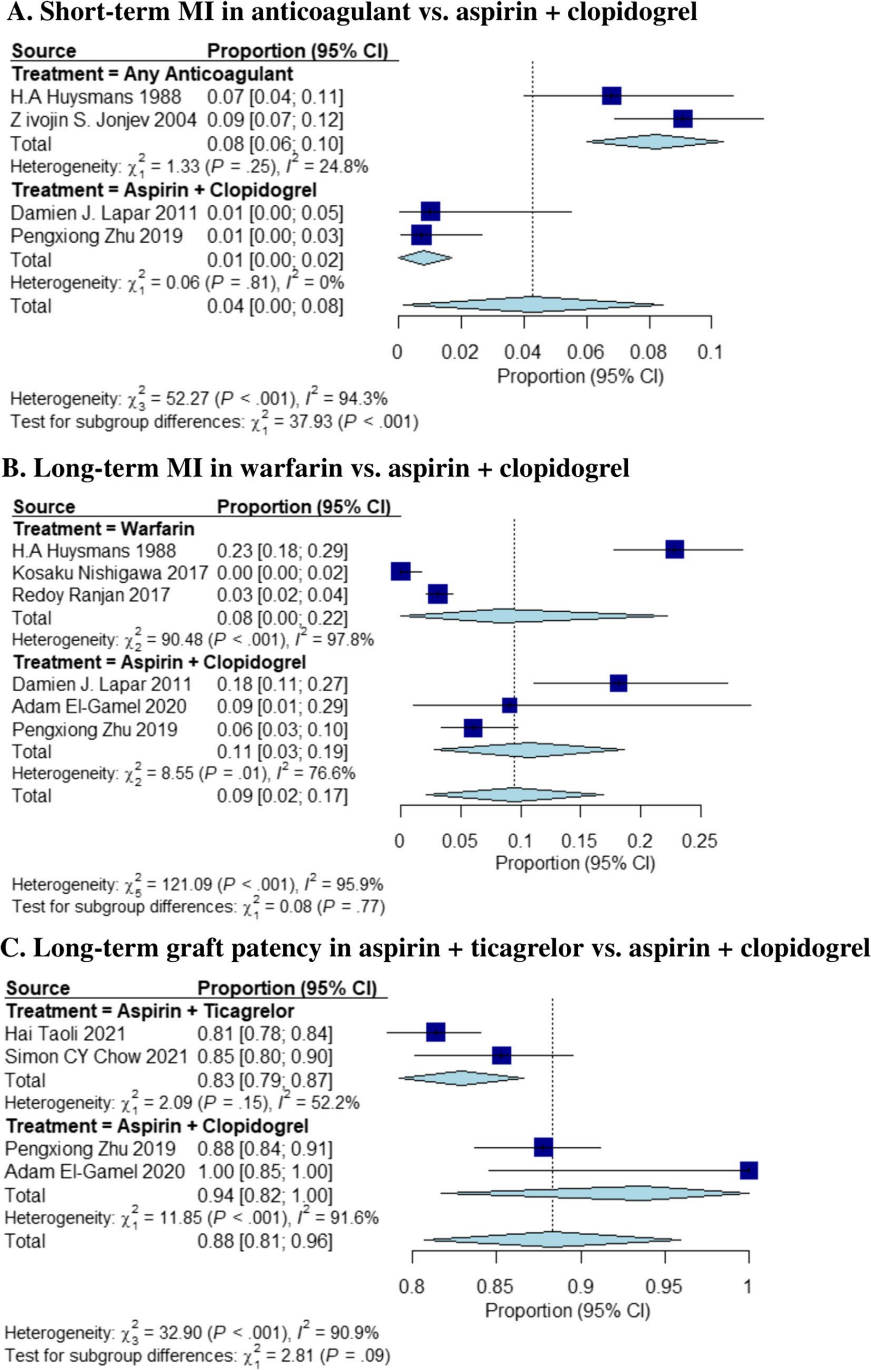
Forest plots showing (**A**) short-term and (**B**) long-term myocardial infarction (MI) in patients receiving anticoagulant versus those on double antiplatelet treatment (DAPT) with aspirin and clopidogrel, and (**C**) long-term graft patency in patients receiving double antiplatelet treatment (DAPT) with aspirin and ticagrelor versus aspirin and clopidogrel

Four studies measured MI events at short-time intervals following endarterectomy in patients treated with either anticoagulant or DAPT with aspirin and clopidogrel [19, 23, 28, 34]. A total of 1225 patients were involved in this assay. A pooled prevalence of 8% (95% CI, 6–10%; *I*^2^ = 24.8%) and 1% (95% CI, 0–2%, *I*^2^ = 0%) was found for anticoagulant and DAPT cohorts respectively. The overall proportion of patients for this analysis was 4% (95% CI, 0–8%; *I*^2^ = 94.3%). Short-term MI was significantly lower in patients receiving aspirin + clopidogrel compared with the anticoagulant cohort (*p* < 0.001) (Fig. 3A).

#### Long-term MI in warfarin vs aspirin + clopidogrel

Six studies measured postoperative MI events at an estimated weighted average of 2.3 years (ranging from 10.5 months to 5.4 years) after CE in patients treated with either warfarin or DAPT with aspirin and clopidogrel [15, 18, 19, 23, 28, 29]. A total of 1824 patients were involved in this assay. A pooled prevalence of 8% (95% CI, 0–22%; *I*^2^ = 97.8%) and 11% (95% CI, 3–19%; *I*^2^ = 76.6%) was found for warfarin and DAPT cohorts respectively. The overall proportion of patients for this analysis was 9% (95% CI, 2–17%; *I*^2^ = 95.9%).

No significant difference in MI prevalence was found between the warfarin cohort and the DAPT group (*p* = 0.77) (Fig. 3B).

#### Long-term graft patency in aspirin + ticagrelor vs aspirin + clopidogrel

Four studies measured mid-term graft patency at a weighted average of 13.4 months (ranging from 12 to 20.2 months) after CE in patients treated with DAPT either with aspirin and ticagrelor or aspirin and clopidogrel [18, 19, 23, 25]. A total of 467 patients were involved in this assay. A pooled prevalence of 83% (95% CI, 79–87%; *I*^2^ = 52.2%) and 94% (95% CI, 82–100%; *I*^2^ = 91.6%) was found for aspirin + ticagrelor and aspirin + clopidogrel cohorts respectively. No significant difference in graft patency was found between these two cohorts (*p* = 0.09) (Fig. 3C).

## Discussion

CE has traditionally been viewed as a “salvage” procedure to achieve the revascularization of ungraftable targets due to its higher morbidity and mortality [12, 14, 16]. In the last 40 years, many papers tried to investigate the reasons underlying these observations without reaching a strong consensus. The main reason lies in the extremely heterogenous population involved in these studies and in the fact that patients who needed CE, compared to those who needed CABG alone, had more severe coronary disease and a more complex comorbidity burden. In this regard, Wang et al. designed a metanalysis on more than 63,000 patients sub-stratifying them according to the preoperative risk profile and found that even in the high-risk population, CE + CABG increased the risk of 30-day mortality by 2.90 times and the risk of postoperative MI by 2.02 times [14].

Damage to the coronary artery endothelium, which increases the risk of thrombosis in the small branches supplying the myocardium, has long been recognized as a primary factor contributing to these observations [4–6]. This mechanism may explain why patients undergoing CE + CABG exhibited worse outcomes in terms of mortality and MI, particularly during the early postoperative period [7, 14, 16]. In addition, the evidence that open-CE + CABG provides better postoperative outcomes in terms of 30-day mortality, postoperative MI, and intra-aortic balloon pump use compared to closed-CE + CABG [35–37] might support this last hypothesis. Moreover, many authors reported that the short- and long-term outcomes seem unrelated to extracorporeal circulation, or the coronary vessel involved in CE [7, 14, 16, 38].

In light of these findings, perioperative antiplatelet/anticoagulant therapy could play a key role in modifying the postoperative course of these patients. As suggested by Soylu et al., the lack of official guidelines about postoperative antiplatelet/anticoagulant therapy introduces a significant source of bias in the analysis of postoperative mortality and morbidity. That variability in treatment strategy can significantly affect postoperative outcomes, creating major differences between the compared populations [39]. In addition, it is important to note that only a few studies precisely report the postoperative therapy; therefore, the number of studies eligible for the meta-analysis is very poor.

Our results suggest that short- and long-term mortalities were not significantly different (*p* = 0.15 and *p* = 0.98 respectively) between patients receiving anticoagulant therapy versus the ones receiving DAPT (Fig. 2A, B). However, the short-term mortality results were aligned with the MI findings, demonstrating the potential for achieving possible significant results by including future investigations in the statistical analysis.

The short-term MI results surprisingly demonstrated that DAPT with aspirin and clopidogrel serves as a safer therapy compared to anticoagulants for post-op CE patients (*p* < 0.001) (Fig. 3A). However, long-term MI results have shown no significant difference between post-op CE patients receiving DAPT with aspirin and clopidogrel and the cohort receiving warfarin treatment (*p* = 0.77) (Fig. 3B).

It is germane to note that in most of the anticoagulation studies warfarin was administered to patients for 3 months, and subsequently the regimen was switched to DAPT for the rest of the course of treatment. Thus, the short-term analysis predominantly reflects patients managed with warfarin-only therapy, thereby enhancing the reliability of the findings within the short-term time frame. Long-term graft patency was also compared between two types of DAPT. Graft patency was found to be comparable between patients treated with aspirin and clopidogrel and those treated with aspirin and ticagrelor (*p* = 0.09) (Fig. 3C).

In light of this and of those previously discussed, we suggest that using a DAPT-only regimen with aspirin and clopidogrel, instead of anticoagulant therapy with warfarin, has potential to serve as a safer postoperative treatment. Regarding the safety of DAPT versus an aspirin-only regimen, a study by Balaj et al. demonstrated superior long-term outcomes in CE patients treated with DAPT compared to those receiving aspirin alone. Specifically, patients in the DAPT group experienced lower rates of mortality and major adverse cardiovascular events following surgery [40].

### Limitations

The main limitation of the present study is related to the lack of big studies or randomized trials on the topic as CE, even though it seems to be having a second birth, has been rarely studied in the last years. Moreover, CE has varied postoperative therapeutic management protocols among the centers. Another limitation encountered, which contributed to the high heterogeneity in our analysis, was the variation in therapeutic regimens and the timing of treatment switches across the studies.

## Conclusions

Postoperative mortality did not differ significantly between the anticoagulant and DAPT cohorts following CE. On the other hand, DAPT seems to lead to a greater reduction in short-term MI rates compared with anticoagulation therapy, making it potentially a better choice as postoperative treatment. However, the reported data demonstrates the potential for achieving possible much more powerful and significant results by including future investigations in the statistical analysis or designing dedicated randomized controlled trials.

## Supplementary Information

Below is the link to the electronic supplementary material.

Supplementary figure S1. Funnel plots showing (A) short-term and (B) long-term mortality in patients receiving anticoagulants versus those on double antiplatelet treatment (DAPT) with aspirin and clopidogrel. (C) short-term and (D) long-term myocardial infarction (MI) in patients receiving anticoagulants versus those on double antiplatelet treatment (DAPT) with aspirin and clopidogrel (PNG 96.6 KB)

High Resolution Image (TIF 264 KB)

Supplementary figure S2. Funnel plots showing long-term graft patency in patients receiving double antiplatelet treatment (DAPT) with aspirin and ticagrelor versus aspirin and clopidogrel (PNG 46.5)

High Resolution Image (TIF 74.3 KB)

Supplementary file3 (DOCX 18 KB)

Supplementary file4 (DOCX 90.7 KB)

Supplementary file5 (MP4 13378 KB)

## Data Availability

The data underlying this article are available in the article and in its online supplementary data.
