# Supplementary material for: Comparative outcomes of anticoagulant versus antiplatelet therapy in coronary endarterectomy: a meta-analysis
Source: Indian J Thorac Cardiovasc Surg. 2026 Jun 16;42(9):1053–65. doi: 10.1007/s12055-026-02221-9 (PMC13438496; doi:10.1007/s12055-026-02221-9)
Supplement: Supplementary file 1 [file 12055_2026_2221_MOESM3_ESM.docx]

**Supplementary File 1 (SF1):** Search strings.

**PubMed:**

"coronary endarterectomy"[tw] AND ("Anticoagulant*"[tw] OR "Anti-coagulant*"[tw] OR "Heparin*"[tw] OR "Warfarain"[tw] OR “apixaban“[tw] OR “dabigatran”[tw] OR ”edoxaban”[tw] OR “Rivaroxaban”[tw] OR “Antiplatelet*”[tw] OR “Anti-platelet*”[tw] OR “aspirin”[tw] OR “clopidogrel”[tw] OR “prasugrel”[tw] OR “ticagrelor”[tw] OR “Dipyridamole”[tw])

……………………………..

**Embase:**

'coronary endarterectomy':ti,ab,kw,de AND ('Anticoagulant*':ti,ab,kw,de OR 'Anti-coagulant*':ti,ab,kw,de OR 'Heparin*':ti,ab,kw,de OR 'Warfarain':ti,ab,kw,de OR 'apixaban':ti,ab,kw,de OR 'dabigatran':ti,ab,kw,de OR 'edoxaban':ti,ab,kw,de OR 'Rivaroxaban':ti,ab,kw,de OR 'Antiplatelet*':ti,ab,kw,de OR 'Anti-platelet*':ti,ab,kw,de OR 'aspirin':ti,ab,kw,de OR 'clopidogrel':ti,ab,kw,de OR 'prasugrel':ti,ab,kw,de OR 'ticagrelor':ti,ab,kw,de OR 'Dipyridamole':ti,ab,kw,de)

……………………………..

**Medline:**

"coronary endarterectomy".tw. AND ("Anticoagulant*".tw. OR "Anti-coagulant*".tw. OR "Heparin*".tw. OR "Warfarain".tw. OR "apixaban".tw. OR "dabigatran".tw. OR "edoxaban".tw. OR "Rivaroxaban".tw. OR "Antiplatelet*".tw. OR "Anti-platelet*".tw. OR "aspirin".tw. OR "clopidogrel".tw. OR "prasugrel".tw. OR "ticagrelor".tw. OR "Dipyridamole".tw.)
