# Supplementary material for: Comparative outcomes of anticoagulant versus antiplatelet therapy in coronary endarterectomy: a meta-analysis
Source: Indian J Thorac Cardiovasc Surg. 2026 Jun 16;42(9):1053–65. doi: 10.1007/s12055-026-02221-9 (PMC13438496; doi:10.1007/s12055-026-02221-9)
Supplement: Supplementary file 2 [file 12055_2026_2221_MOESM4_ESM.docx]

**Supplementary table 1 (ST1):** Table listing the additional papers that were screened for eligibility for inclusion in the meta-analysis.

| Leading author | Title | Doi/PMID |
| --- | --- | --- |
| Abdul R Abid et al., (2009) | Hospital Outcome of Coronary Artery Bypass Grafting and Coronary Endarterectomy | doi:10.1177/0218492309102609 |
| Brenowitz JB et al., (1988) | Results of coronary artery endarterectomy and reconstruction | PMID: 3257284. |
| D. Craig Miller et al., (1981) | Long-term clinical assessment of the efficacy of adjunctive coronary endarterectomy | <https://doi.org/10.1016/S0022-5223(19)37655-X> |
| Damien J. LaPar et al., (2011) | The Impact of Coronary Artery Endarterectomy on Outcomes During Coronary Artery Bypass Grafting | doi:10.1111/j.1540-8191.2011.01247.x. |
| Erdinc Soylu et al., (2014) | Adjunct coronary endarterectomy increases myocardial infarction and early mortality after coronary artery bypass grafting: a meta-analysis | doi:10.1093/icvts/ivu157 |
| Gale et al., (1977) | Right coronary endarterectomy: a procedure with increased risk of perioperative infarction | doi:10.1111/j.1445-2197.1977.tb04338.x |
| George Asimakopoulos et al., (1993) | Does Coronary Endarterectomy Adversely Affect the Results of Bypass Surgery? | doi:10.1111/j.1540-8191.1993.tb00577.x |
| H.A. Huysmans et al., (1988) | Treatment of Peripheral Coronary Artery Disease by Endarterectomy | doi:10.1159/000415609 |
| ImranHussain et al., (2008) | In hospital outcome of patients undergoing coronaray endarterectomy: comparison between off-pump vs on pump CABG | PMID: 19024182 |
| James J. Livesay et al., (1988) | Early and Late Results of Coronary Endarterectomy in 3,369 Patients | doi:10.1159/000415611 |
| John J. Kelly et al., (2022) | Coronary Endarterectomy: Analysis of The Society of Thoracic Surgeons Adult Cardiac Surgery Database | <https://doi.org/10.1016/j.athoracsur.2022.01.067> |
| M. Laxman Kamath et al., (1981) | Patency and Flow Response in Endarteredomized Coronary Arteries | <https://doi.org/10.1016/S0003-4975(10)61313-6> |
| Marie-Paule Larock et al., (1987) | Usefulness of Thromboendarterectomy in with Coronary Artery bypass grafting | doi:10.1016/0002-9149(87)90346-8 |
| Mohammad Hassan Nemati et al., (2015) | Outcome and Graft Patency in Coronary Artery Bypass Grafting with Coronary Endarterectomy | http://dx.doi.org/10.5090/kjtcs.2015.48.1.13 |
| Nevzat Erdil et al., (2002) | Closed Endarterectomy for Diffuse Right Coronary Artery Disease: Early Results with Ang iog raph ic Controls | doi: 10.1111/j.1540-8191.2001.tb01137.x |
| O Taşdemir et al., (1996) | Long-term results of reconstructions of the left anterior descending coronary artery in diffuse atherosclerotic lesions | doi: 10.1016/S0022-5223(96)70061-2 |
| Oz M. Shapira et al., (1999) | Improved Clinical Outcomes in Patients Undergoing Coronary Artery Bypass Grafting With Coronary Endarterectomy | doi:10.1016/s0003-4975(99)01050-4 |
| Paul J.Walter et al., (1988) | Endarterectomy in Patients with Diffuse Coronary Artery Disease | doi: 10.1159/000415613 |
| Ravindranath Tiruvoipati et al., (2005) | Coronary Endarterectomy: Impact on Morbidity and Mortality When Combined With Coronary Artery Bypass Surgery | doi: 10.1016/j.athoracsur.2004.12.041 |
| Shuli Silberman et al., (2002) | Does Coronary Endarterectomy Increase the Risk of Coronary Bypass? | doi: 10.1111/j.1540-8191.2001.tb01138.x |
| Srikrishna Sirivella et al., (2005) | Results of Coronary Artery Endarterectomy and Coronary Artery Bypass Grafting for Diffuse Coronary Artery Disease | doi:10.1016/j.athoracsur.2005.05.034 |
| Toshihiro Fukui et al., (2010) | Extensive Reconstruction of the Left Anterior Descending Coronary Artery With an Internal Thoracic Artery Graft | doi:10.1016/j.athoracsur.2010.10.002 |
| Yasuyuki Kato et al., (2012) | Results of Long Segmental Reconstruction of Left Anterior Descending Artery Using Left Internal Thoracic Artery | doi:10.1016/j.athoracsur.2011.12.059 |
| Zhibing Qiu et al., (2014) | Comparison of off-pump and on-pump coronary endarterectomy for patients with diffusely diseased coronary arteries: early and midterm outcome | doi:10.1186/s13019-014-0186-5 |
| Ziyad M Binsalamah et al., (2013) | Mid-Term Outcome and Angiographic Follow-Up of Endarterectomy of the Left Anterior Descending Artery in Patients Undergoing Coronary Artery Bypass Surgery | doi: 10.1111/jocs.12230 |

**Supplementary Table 2 (ST2):** Table listing the perioperative data of included studies

| Year | Leading Author | Patients | Age | Male sex (%) | Hypertension | Diabetes Mellitus | Smoking | Obesity/Average BMI | Hyperlipidemia | COPD | Family history of CAD | Chronic renal failure | Stable angina | Unstable angina | Arrythmia | MI in general | Previous PCI | Euro score | SYNTAX score | Cerebrovascular event | Pre-op LVEF | NYHA class III or IV | Prior cardiac surgery | Triple vessel disease | History of congestive heart failure | Peripheral vascular disease | Left main disease |
| --- | --- | --- | --- | --- | --- | --- | --- | --- | --- | --- | --- | --- | --- | --- | --- | --- | --- | --- | --- | --- | --- | --- | --- | --- | --- | --- | --- |
| 2009 | Abdul R Abid et al. | 50 | 53.56 ± 10.16 | 90% | 46% | 36% | 44% | 14% | 14% | NR | 34% | NR | 46% | 24% | NR | 30% | NR | NR | NR | NR | NR | NR | NR | NR | NR | NR | NR |
| 2020 | Adam El-Gamel et al. | 22 | 64 (60-84) | 72% | 36.36% | 45.45% | NR | NR | NR | NR | NR | NR | NR | NR | NR | NR | NR | 2.1 | NR | NR | 40% | NR | NR | NR | NR | NR | NR |
| 2011 | Damien J. Lapar et al. | 99 | 62.2 ± 10.4 | 70.7% | 85.9% | 47.5% | 54.5% | NR | 90.9% | NR | 58.6% | 8.1% | 62.6% | | 17.2% | 57.6% | 20.2% | NR | NR | 21.2% | NR | NR | 3% | NR | NR | 17.2% | NR |
| 2020 | Dimitar Kyuchukov et al. | 24 | 59.86 ± 9.88 | 66.7% | 95.8% | 54.2% | 83.3% | 29.2% | NR | 16.6% | NR | NR | NR | NR | NR | NR | NR | 3.9 | NR | NR | 41.6 % | NR | NR | NR | NR | NR | NR |
| 1988 | H.A Huysmans et al. | 250 | 56.8 (34-81) | 88% | NR | NR | NR | NR | NR | NR | NR | NR | NR | NR | NR | NR | NR | NR | NR | NR | NR | NR | NR | NR | NR | NR | NR |
| 2021 | Hai Tao Li et al. | 94 | 62.6 ± 7.9 | 63.20% | 53.7% | 48.4% | 47.4% | NR | NR | NR | NR | 7.4% | 23.4% | | NR | 10.6% | 37.8% | NR | NR | 4.3% | 54.8 ± 6.7 | 14.7% | NR | NR | NR | NR | NR |
| 2020 | Hua Yan et al. | 67 | 61.4 | 74.6% | 67.2% | 44.8% | 58.2% | 25.5 | 83.6% | NR | NR | 3.0% | NR | NR | NR | 47.8% | 14.9% | NR | NR | 16.4% | 62.0% | 43.3% | 1.5% | 73.1% | NR | NR | NR |
| 2017 | Kosaku Nishigawa et al. | 205 | 66.28 | 85.85% | 73.17% | 60% | 52.2% | NR | 70.2% | 0.5% | NR | 7.8% | NR | 21% | NR | 40.5% | 38.0% | NR | NR | 10.7% | 54.7% | NR | NR | 84.4% | 18.5% | 9.8% | 23.4% |
| 2021 | Kosaku Nishigawa et al. | 233 | 65.9 ± 9.5 | 85.8% | NR | NR | NR | NR | NR | NR | NR | NR | NR | NR | NR | NR | NR | NR | NR | NR | NR | NR | NR | NR | NR | NR | NR |
| 2016 | Marco Russo et al. | 52 | 66.6 ± 7.8 | 67% | 90% | 15% | 25% | 19% | 42% | 7% | NR | 7% | NR | NR | NR | 28% | NR | NR | NR | NR | 51.1 % ± 9.87 | NR | NR | NR | NR | 17% | 28% |
| 2015 | Mohammad Nemati et al. | 84 | 61.5 ± 9.27 | 65.47% | 75.9% | 48.2% | 39.5% | 25.6 ± 4.1 | NR | NR | 61.3% | NR | NR | NR | NR | NR | NR | NR | NR | NR | 46.6 ± 14.4 | NR | NR | NR | NR | NR | 8.3% |
| 2019 | Pengxiong Zhu et al. | 270 | 64.98 | 85.55% | 76.1% | 24.3% | 24.5% | NR | 10.9% | 2.9% | 5.4% | NR | NR | NR | NR | 30.4% | NR | NR | NR | 18.2% | 59.3% | NR | NR | NR | NR | NR | NR |
| 2017 | Redoy Ranjan et al. | 1000 | 61.5 ± 5.5 | 78.2% | 82.1% | 35.8% | 71.9% | NR | 76.5% | NR | 55.8% | NR | NR | NR | NR | 72.4% | NR | 5.9 ± 1.7 | NR | NR | NR | 66.1% | NR | NR | NR | NR | NR |
| 2022 | Sharaf-Eldin Shehada et al., (DAT group) | 174 | 66.7 ± 9.3 | 88% | NR | 38.3% | 35.3% | 27.4 ± 4.1 | 62.6% | 11.6% | NR | NR | NR | NR | NR | 37.7% | NR | NR | 33.1 ± 12 | 7.7% | NR | 33.1% | 5.5% | 93% | NR | 16.3% | NR |
| 2021 | Simon CY Chow et al. | 81 | 61.9 ± 9.29 | 82.7% | 80.2% | 57.3% | 55.6% | NR | 58.0% | NR | NR | NR | NR | NR | NR | 70.4% | NR | NR | NR | NR | 51% ± 12.5% | 17.3% | NR | 95.1% | NR | NR | 48.1% |
| 2005 | Toshihiro Fukui et al. | 11 | 64 (43-75) | 72.72% | NR | NR | NR | NR | NR | NR | NR | NR | NR | NR | NR | NR | 100% | NR | NR | NR | NR | NR | NR | 81.8% | NR | NR | NR |
| 2012 | Yasuyuki Kato et al. | 37 | 61.9 ± 10.4 | 68% | 65% | 59% | 41% | NR | 57% | NR | NR | NR | NR | NR | NR | 65% | 43% | NR | NR | 18.9% | 51.7 ± 13% | NR | NR | NR | NR | 5.4% | 24% |
| 2004 | Z˘ ivojin S. Jonjev et al. | 606 | 55.8 ± 6.2 | 84.98% | 56.3% | 55.8% | 58.7% | NR | 67.6% | NR | 43.2% | NR | NR | 64.5% | NR | 69.5% | NR | NR | NR | NR | 40.3% | NR | NR | 56.8% | NR | NR | 8.58% |
| 2013 | Ziyad M. et al. | 58 | 67.3 ± 10.6 | 90% | 69% | NR | NR | NR | NR | 14% | NR | 3% | NR | NR | NR | NR | NR | NR | NR | NR | 49.1% ± 9 | NR | NR | NR | 10% | 12% | 17% |

Data is reported either in Percentage or mean ± SD or median (range).; **BMI:** Body Mass Index; **CAD:** Coronary Artery Disease; **CCS:** Canadian Cardiovascular Society; **COPD:** Chronic Obstructive Pulmonary Disease; **LVEF:** Left Ventricular Ejection Fraction; **MI:** Myocardial Infarction; **NYHA:** New York Heart Association; **NR:** Not Reported; **PCI:** Percutaneous Coronary Intervention; **SYNTAX:** Synergy between PCI with Taxus and Cardiac Surgery

**Supplementary Table 3 (ST3):** Table listing the intraoperative data of included studies

| Year | Leading Author | Patients | Off-pump | No. of distal anastomosis in average | Aortic cross-clamp (min) | CPB (min) | RCA graft exclusively | LAD graft exclusively | RCA + Diagonal graft | RCA + OM graft | RCA + PDA graft | RCA + LAD graft | OM graft | PDA graft | LCX graft | LAD + Diagonal graft | Diagonal exclusively | IMA in general | Right IMA | Left IMA | Right radial artery | Saphenous | Arterial graft in general | Two grafts | Three grafts | No. of vessels bypassed | One CEA | Two CEA | Three CEA | No. of endarterectomized vessels | LAD Endarterectomy | OM endarterectomy | LCX endarterectomy | RCA/PDA endarterectomy | Diagonal endarterectomy |
| --- | --- | --- | --- | --- | --- | --- | --- | --- | --- | --- | --- | --- | --- | --- | --- | --- | --- | --- | --- | --- | --- | --- | --- | --- | --- | --- | --- | --- | --- | --- | --- | --- | --- | --- | --- |
| 2009 | Abdul R Abid et al. | 50 | 17 (34%) | NR | NR | NR | 30 (60%) | NR | NR | NR | NR | NR | 34 (68%) | 11 (22%) | 5 (10%) | NR | 19 (38%) | NR | 0 (0%) | 50 (31.4%) | 0 (0%) | 109 (68.6%) | NR | NR | NR | 159 | NR | NR | NR | 56 | 21 (42%) | 1 (2%) | 7 (14%) | 22 (44%) | 3 (6%) |
| 2020 | Adam El-Gamel et al. | 22 | NR | NR | 65 ± 5 | 77.2 ± 4 | NR | NR | NR | NR | NR | NR | NR | NR | NR | NR | NR | NR | NR | NR | NR | NR | NR | NR | NR | NR | NR | NR | NR | NR | NR | NR | NR | NR | NR |
| 2011 | Damien J. Lapar et al. | 99 | NR | NR | 95.6 ± 2.8 | 121.8 ± 3.8 | NR | NR | NR | NR | NR | NR | NR | NR | NR | NR | NR | 98 (99%) | 0.5% | NR | 1.5% | NR | NR | 12 (12.1%) | 87 (87.9%) | 285 | NR | NR | NR | NR | 12.1% | 3% and RCA + OM: 1.0% | 1.0% | 80.0% RCA and 2.0% PDA | 1.0% RCA+ Diagonal |
| 2020 | Dimitar Kyuchukov et al. | 24 | NR | 2.9 ± 0.43 | 44.6 ± 3.4 | 74.2 ± 10.2 | 21 (87.5%) | 2 (8.3%) | NR | NR | NR | NR | NR | NR | 1 (4.2%) | NR | NR | NR | NR | NR | NR | NR | NR | NR | NR | NR | NR | NR | NR | NR | NR | NR | NR | NR | NR |
| 1988 | H.A Huysmans et al. | 250 | NR | NR | NR | NR | NR | NR | NR | NR | NR | NR | NR | NR | NR | NR | NR | NR | NR | NR | NR | NR | NR | NR | NR | NR | NR | NR | NR | 339 | 52 (15.3%) | 25 (7.4%) | 0 (0%) | 147 (43.4%) RCA and 43 (12.7%) PDA | 37 (10.9%) |
| 2021 | Hai Tao Li et al. | 94 | 94 (100%) | NR | NR | NR | NR | NR | NR | NR | NR | NR | NR | NR | NR | NR | NR | NR | NR | NR | NR | NR | NR | NR | NR | NR | NR | NR | NR | NR | NR | NR | NR | NR | NR |
| 2020 | Hua Yan et al., (AC group) | 67 | 40 (59.7%) | 3.5 ± 0.8 | NR | NR | NR | NR | NR | NR | NR | NR | NR | NR | NR | NR | NR | 67 (100%) | NR | NR | NR | NR | NR | NR | NR | NR | NR | NR | NR | NR | 26 (38.8%) | None | 4 (6%) | RCA: 33 (49.3%) | Diagonal + intermediate: 4 (6%) |
| 2017 | Kosaku Nishigawa et al. | 205 | 205 (100%) | NR | NR | NR | NR | NR | NR | NR | NR | NR | NR | NR | NR | NR | NR | NR | NR | NR | NR | NR | NR | NR | NR | NR | NR | NR | NR | NR | NR | NR | NR | NR | NR |
| 2021 | Kosaku Nishigawa et al. | 233 | 99.6% | 4.5 ± 1.3 | NR | NR | NR | NR | NR | NR | NR | NR | NR | NR | NR | NR | NR | NR | NR | NR | NR | NR | NR | NR | NR | NR | NR | NR | NR | NR | NR | NR | NR | NR | NR |
| 2016 | Marco Russo et al., (DAT group) | 52 | 6 (11%) | 2.9 ± 0.7 | 58 ± 14 | 96 ± 21 | NR | NR | NR | NR | NR | NR | NR | NR | NR | NR | NR | Bilateral IMA:6 (11%) | NR | 51 (98.1%) | NR | 48 (92%) | NR | NR | NR | NR | NR | NR | NR | NR | 40 (76%) | 3 (5%) | NR | RCA/RPD: 10 (19%) | 1 (2%) |
| 2015 | Mohammad Hassan Nemati et al. | 84 | 0 (0%) | 4.11 ± 0.74 | NR | NR | NR | NR | NR | NR | NR | NR | NR | NR | NR | NR | NR | NR | NR | NR | NR | NR | NR | NR | NR | 113 | NR | NR | NR | 29 | 12 (41.4%) | NR | NR | RCA: 8 (27.6%) | NR |
| 2019 | Pengxiong Zhu et al. | Peri-op: 270  Mid-term: 248 | 100% | NR | NR | NR | 217 (68%) | 60 (19%) | NR | NR | NR | NR | NR | NR | 42 (13%) | NR | NR | NR | NR | NR | NR | NR | NR | NR | NR | NR | NR | NR | NR | NR | 46 (17%) | NR | 30 (11%) | RCA: 194 (72%) | NR |
| 2017 | Redoy Ranjan et al. | 1000 | 872 (87.2%) | 3.3 | NR | NR | NR | NR | NR | NR | NR | NR | NR | NR | NR | NR | NR | NR | 428 (42.8%) | 100% | NR | NR | NR | 112 (11.2%) | 538 (53.8%) | NR | NR | NR | NR | 1198 | 432 (43.2%) and LAD+RCA: 57 (5.7%) | 49 (4.9%) | NR | RCA: 164 (16.4%) and PDA: 89 (8.9%) | 266 (26.6%) and LAD+ Diagonal: 24 (2.4%) and RCA + Diagonal: 18 (1.8%) |
| 2022 | Sharaf-Eldin Shehada et al., (DAT group) | 174 | NR | 4.3 ± 1.1 | 84.3 ± 19.2 | NR | NR | NR | NR | NR | NR | NR | NR | NR | NR | NR | NR | NR | NR | NR | NR | 249 (63.2%) | 145 (36.8%) | NR | NR | 394 | 269 (82.5%) | 47 (14.4%) | 9 (2.8%) | NR | 174 (44.2%) | NR | 67 (17%) | RCA: 153 (38.8%) | NR |
| 2021 | Simon CY Chow et al. | 81 | NR | NR | NR | NR | NR | NR | NR | NR | NR | NR | NR | NR | NR | NR | NR | NR | NR | NR | NR | 77 (69.4%) | 34 (30.6%) | NR | NR | 111 | NR | NR | NR | 41 | 9 (22%) | 6 (14.6%) | None | 23 (56.1%) | 3 (7.3%) |
| 2005 | Toshihiro Fukui et al. | 11 | 63.6% | 3.8 | NR | NR | NR | NR | NR | NR | NR | NR | NR | NR | NR | NR | NR | NR | NR | NR | NR | NR | NR | NR | NR | NR | NR | NR | NR | NR | NR | NR | NR | NR | NR |
| 2012 | Yasuyuki Kato et al. | 37 | 4.0% | 4.0 ± 1.2 | 143 ± 31 | 198 ± 53 | NR | NR | NR | NR | NR | NR | NR | NR | NR | NR | NR | NR | NR | 5 (4%) | NR | NR | NR | NR | NR | NR | NR | NR | NR | NR | NR | NR | NR | NR | NR |
| 2004 | Z˘ ivojin S. Jonjev et al. | 606 | NR | NR | NR | NR | NR | NR | NR | NR | NR | NR | NR | NR | NR | NR | NR | NR | NR | NR | NR | NR | NR | NR | NR | NR | NR | NR | NR | NR | NR | NR | NR | NR | NR |
| 2013 | Ziyad M. et al. | 58 | NR | 5.3 ± 1.4 | 127 ± 24.2 | 149.7 ± 28.6 | NR | NR | NR | NR | NR | NR | NR | NR | NR | NR | NR | NR | NR | NR | NR | NR | NR | NR | NR | NR | NR | NR | NR | NR | NR | NR | NR | NR | NR |

Data is reported either in N. (percentage) or mean ± SD.; **APT:** Anti platelet therapy; **CEA:** Coronary endarterectomy; **CPB:** Cardiopulmonary Bypass; **IMA:** Internal Mammary Artery; **LAD:** Left Anterior Descending; **LCX:** Left Circumflex; NR: Not Reported; **OM:** Obtuse Marginal; **PDA:** Posterior Descending Artery; **RCA:** Right Coronary Artery

**Supplementary Table 4 (ST4):** Table listing the postoperative data of included studies

| Year | Leading Author | Patients | Hospital death | Cardiac related mortality | Postoperative mortality | Postoperative LVEF (%) | Perioperative MI | Overall postoperative MI | Atrial fibrillation | Arrhythmia | Cerebrovascular accident | ICU stay (days) | Hospital stay (days) | Ventricular arrythmias | Stroke in general | Hemorrhagic stroke | Ischemic stroke | Renal impairment | Blood transfusion (mls or percentage) | Tamponade | Graft occlusion | Pulmonary embolism | Prolonged ventilation | Bleeding events | MACCE | Need for revascularization | Hospitalization for heart failure | Cardiac arrest | Need for IABP | Need for ECMO | Need for reintubation | Postoperative dialysis | Neurological complications | Back to theater |
| --- | --- | --- | --- | --- | --- | --- | --- | --- | --- | --- | --- | --- | --- | --- | --- | --- | --- | --- | --- | --- | --- | --- | --- | --- | --- | --- | --- | --- | --- | --- | --- | --- | --- | --- |
| 2009 | **Abdul R Abid et al.** | 50 | 1 (2%) | NR | NR | NR | NR | 2 (4%) | 3 (6%) | NR | 1 (2%) | 1.78 ± 1 | 12.6 ±5.7 | 1 (2%) | NR | NR | NR | NR | NR | NR | NR | NR | NR | NR | NR | NR | NR | NR | NR | NR | NR | NR | NR | NR |
| 2020 | **Adam El-Gamel et al.** | 22 | 0 (0%) | NR | NR | NR | NR | 2 (9%) | NR | NR | NR | 0.37 (0-5) | 6.3 (4-19) | 3 (3%) | 0 (0%) | NR | NR | 1 (4%) | 350 (0-3134) | NR | NR | NR | NR | NR | NR | NR | NR | NR | NR | NR | NR | NR | NR | NR |
| 2011 | **Damien J. Lapar et al.** | 99 | NR | NR | 4 (4%) | NR | 1 (1%) | 18 (18.1%) | 14 (14.1%) | NR | NR | NR | NR | NR | 3 (3%) | NR | NR | 5 (5.1%) | NR | 2 )2%( | 0 (0%) | 1 (1%) | NR | NR | NR | NR | NR | 4 (4%) | 15.2% | NR | 5 (5.1%) | 4 (4%) | NR | NR |
| 2020 | **Dimitar Kyuchukov et al.** | 24 | NR | NR | 4.1% | 41.05 ± 10.5 | NR | NR | NR | NR | NR | NR | NR | NR | NR | NR | NR | NR | NR | NR | NR | NR | NR | NR | NR | NR | NR | NR | NR | NR | NR | NR | NR | NR |
| 1988 | **H.A Huysmans et al.** | 250 | 1.6% | NR | NR | NR | NR | 6.8% | NR | NR | NR | NR | NR | NR | NR | NR | NR | 4.8% | NR | NR | NR | NR | NR | NR | NR | NR | NR | NR | 10 (4%) | NR | NR | NR | NR | NR |
| 2021 | **Hai Tao Li et al.** | 94 | NR | NR | 7 (704%) | NR | NR | 22 (23.4%) | NR | NR | 4 (4.3%) | NR | NR | NR | NR | NR | NR | NR | NR | NR | NR | NR | NR | NR | 17 (18.1%) | 0 (0%) | NR | NR | NR | NR | NR | NR | NR | NR |
| 2020 | **Hua Yan et al., (AC group)** | 67 | 0 (0%) | NR | NR | 60 (58-63) | 1 (1.5%) | 2 (3%) | 11 (16.4%) | NR | NR | 44 (19.7%) | NR | NR | NR | 0 (0%) | 1 (1.5%) | NR | 9 (13.4%) | 1 (1.5%) | NR | NR | NR | NR | 2 (3%) | 0 (0%) | NR | NR | NR | NR | NR | NR | NR | NR |
| 2017 | **Kosaku Nishigawa et al.** | 205 | NR | 5 (2.4%) | 29 (14.1%) | NR | NR | NR | NR | NR | 16 (7.8%) | NR | NR | NR | NR | NR | NR | NR | NR | NR | NR | NR | NR | NR | NR | 33 (16.1%) | NR | NR | NR | NR | NR | NR | NR | NR |
| 2021 | **Kosaku Nishigawa et al.** | 233 | NR | NR | NR | NR | NR | NR | NR | NR | NR | NR | NR | NR | 8 (3.4%) | NR | NR | NR | NR | NR | NR | NR | NR | NR | NR | NR | NR | NR | NR | NR | NR | NR | NR | NR |
| 2016 | **Marco Russo et al., (DAT group)** | 52 | 2 (4%) | NR | NR | 51 ± 8.7 | NR | 6 (11%) | 14 (26%) | NR | NR | NR | 6.5 ± 2.6 | NR | NR | NR | NR | 1 (2%) | NR | NR | NR | NR | NR | 1 (2%) | NR | NR | NR | NR | NR | NR | NR | NR | 0 (0%) | NR |
| 2015 | **Mohammad Hassan Nemati et al.** | 84 | 1.25 % | NR | NR | 52.06 ± 9.22 | NR | NR | NR | NR | NR | 2.78 ± 1.09 | 5.49 ± 1.43 | NR | NR | NR | NR | NR | NR | NR | NR | NR | NR | NR | NR | NR | NR | NR | NR | NR | NR | NR | NR | NR |
| 2019 | **Pengxiong Zhu et al.** | Peri-op: 270  Mid-term: 248 | 4 (1.5%) | NR | mid-term: 22 (8.9%) | NR | 2 (0.7%) | Mid-term: 15 (6.0%) | NR | NR | NR | NR | NR | NR | Peri-op: 4 (1.5%), Mid-term:24 (9.7%) | NR | NR | NR | NR | NR | 39/274 (14.2%) | NR | NR | NR | Peri-op: 10 (3.7%), Mid-term: 45 (18.1%) | NR | NR | NR | NR | NR | NR | NR | NR | NR |
| 2017 | **Redoy Ranjan et al.** | **1000** | **19 (1.9%)** | **NR** | **>6 months:11 (1.1%)** | **NR** | **NR** | **31 (3.1%)** | **157 (15.7%)** | **NR** | **NR** | **1.52 ± 0.28** | **10 ± 1** | **NR** | **NR** | **NR** | **NR** | **23 (2.3%)** | **1.5 ± 0.5** | **NR** | **NR** | **NR** | **NR** | **NR** | **NR** | **NR** | **NR** | **NR** | **8 (0.8%)** | **NR** | **NR** | **NR** | **14 (1.4%)** | **7 (0.7%)** |
| 2022 | **Sharaf-Eldin Shehada et al., (DAT group)** | **174 (this data was reported in n=326)** | **13 (4%)** | **11 (3.3%)** | **NR** | **NR** | **NR** | **8 (2.4%)** | **NR** | **NR** | **NR** | **2.4 ± 2.1** | **NR** | **NR** | **8 (2.4%)** | **NR** | **3 (0.9%)** | **NR** | **NR** | **NR** | **NR** | **NR** | **NR** | **18 (5.5%)** | **In n=174 group:**  **47 (27%)** | **NR** | **NR** | **NR** | **20 (6.1%)** | **5 (1.5%)** | **23 (7%)** | **22 (6.7%)** | **NR** | **NR** |
| 2021 | **Simon CY Chow et al.** | **81** | **NR** | **In 1year: 4 (4.9%)** | **In 30days: 2(2.5), In 1year:5 (6.2%)** | **NR** | **6 (7.4%)** | **NR** | **21 (25.9%)** | **NR** | **NR** | **NR** | **NR** | **NR** | **2 (2.4%)** | **NR** | **1(1.2%)** | **NR** | **NR** | **NR** | **NR** | **NR** | **NR** | **NR** | **9 (11.1%)** | **3 (3.7%)** | **NR** | **NR** | **NR** | **NR** | **NR** | **Peri-op: 1(1.2%)** | **NR** | **NR** |
| 2005 | **Toshihiro Fukui et al.** | **11** | **0 (0%)** | **NR** | **NR** | **58% ± 8.3** | **1 (9.1%)** | **NR** | **NR** | **0 (0%)** | **NR** | **NR** | **11.3 ± 1.3** | **NR** | **0 (0%)** | **0 (0%)** | **0 (0%)** | **0 (0%)** | **NR** | **NR** | **NR** | **NR** | **NR** | **0 (0%)** | **NR** | **NR** | **NR** | **NR** | **NR** | **NR** | **NR** | **NR** | **NR** | **NR** |
| 2012 | **Yasuyuki Kato et al.** | **37** | **1 (2.7%)** | **NR** | **NR** | **NR** | **NR** | **NR** | **NR** | **1 (2.7%)** | **NR** | **NR** | **21.9 ± 12** | **NR** | **2 (5.4%)** | **NR** | **NR** | **NR** | **NR** | **NR** | **1/35 (3%)** | **NR** | **NR** | **0 (0%)** | **NR** | **NR** | **NR** | **NR** | **NR** | **NR** | **NR** | **NR** | **NR** | **NR** |
| 2004 | **Z˘ ivojin S. Jonjev et al.** | **606** | **NR** | **20 (3.3%)** | **30days: 28 (4.6%)** | **NR** | **NR** | **NR** | **NR** | **57 (9.4%)** | **NR** | **NR** | **NR** | **NR** | **NR** | **NR** | **NR** | **NR** | **NR** | **NR** | **NR** | **NR** | **NR** | **24 (4.0%)** | **NR** | **NR** | **NR** | **NR** | **NR** | **NR** | **NR** | **NR** | **NR** | **NR** |
| 2013 | **Ziyad M. et al.** | **58** | **1 (1.7%)** | **NR** | **NR** | **NR** | **NR** | **1 (2%)** | **NR** | **20 (34%)** | **NR** | **1.2 ± 0.3** | **10.0 ± 4.3** | **NR** | **NR** | **NR** | **NR** | **1 (2%)** | **NR** | **NR** | **NR** | **NR** | **NR** | **2 (3%)** | **NR** | **NR** | **NR** | **NR** | **4 (7%)** | **NR** | **NR** | **NR** | **1 (2%)** | **NR** |

Data is reported either in N. (percentage) or mean ± SD or median (range).;**ECMO:** Extracorporeal Membrane Oxygenation; **ICU:** Intensive Care Unit; **IABP:** Intra-Aortic Balloon Pump; **LVEF:** Left Ventricular Ejection Fraction; **MACCE:** Major Adverse Cardiac and Cerebrovascular Events; **MI:** Myocardial Infarction; **NR:** Not Reported

**Supplementary Table 5 (ST5):** Table listing the surgical methodology, graft type, and myocardial infarction definition of included studies

| Study | Graft type | MI definition | Endarterectomy technique |
| --- | --- | --- | --- |
| Abdul R Abid et al., (2009) | Saphenous, LIMA (LIMA always anastomosed to the LAD) | Peri-op MI was characterized by ECG changes (lack of progressive R waves rise of Q waves, or intraventricular conduction abnormalities), along with the elevated levels of cardiac troponin or creatine kinase-MB or emergence of irregularities in myocardial wall movement in Echo within 48h post-op. | An incision of limited extent was created at the designated anastomosis site, allowing for the he separation of the atherosclerotic plaque both in upstream and downstream through the use of an endarterectomy spatula, facilitating extraction via a traction technique. In the case of the LAD, care was taken to restrict the proximal endarterectomy to ensure the preservation of septal perforators. For other vessels, the division of plaque occurred 2 mm proximal to the incision, serving to mitigate potential competitive flow issues. Beating heart procedures simplified carotid endarterectomy due to natural counter-traction The core underwent examination for a smooth tapering end, and additional incisions were made as needed, closed with a vein patch. LIMA was anastomosed distally end-to-side by 7/0 Prolene; vein grafts were used if LIMA size or arteriotomy exceeded 2 cm. |
| Adam El-Gamel et al., (2020) | The LITA was harvested in a non-skeletonized manner, and the saphenous vein was procured for utilization as a patch in all cases within the FMJ-LAD group. | MI was characterized by the persistent emergence of novel electrocardiographic (ECG) alterations concomitant with elevated levels of cardiac enzymes, in accordance with the universal criteria established for MI. All patients underwent continuous daily monitoring of 12-lead ECGs for a duration of five days post-op. | Manual CEA were exclusively conducted for the FMJ-LAD group using the open method, employing a WatsonCheyne dissector to create a dissection plane between the media layer and the central core of the atheromatous plaque that had been stented. CEA was extended as necessary, ranging from 2.5 to 6 cm. If the distal atheroma lacked tapering, the arteriotomy was elongated to achieve a satisfactory outcome. Subsequently, retrograde cardioplegia was administered to minimize distal LAD debris, thus reducing the likelihood of distal runoff obstruction. After the removal of the stent, a venous graft was utilized to reconstruct the LAD, and an LITA to LAD anastomosis was performed when deemed necessary. Subsequently, the standard CABG procedure was finalized. |
| Damien J. Lapar et al., (2011) | Saphenous vein and IMA | Perioperative MI was specifically defined as either a post-op CK-MB level exceeding five times the upper limit of normal within the initial 24 hours or the presence of one or more of the subsequent criteria during the same hospitalization period exceeding 24 hours: progressive ST-segment elevations, emergence of new Q waves in two consecutive ECG leads, the onset of new left bundle branch block, or a CK-MB level surpassing three times the upper limit of normal. | During right CEA an arteriotomy was created at the anastomosis site. The extraction of the plaque was accomplished using an eversion traction method. If the plaque's endpoint lacked a tapered, feathered appearance, the arteriotomy was extended. The terminal portion of a Saphenous graft prepared with a widened end was utilized both for revascularization and the closure of the arteriotomy. Similarly, in cases of CEA involving the LAD, an analogous open eversion method was utilized. Due to the typically extended LAD arteriotomy, our practice was to close it with a saphenous before conducting bypass with the IMA. |
| Dimitar Kyuchukov et al., (2020) | In the revascularization of the LAD, the ITA was utilized, while other vessels were revascularized utilizing the saphenous. | Post-op ECG alterations (such as new Q waves or ST-T segment changes), a fivefold or greater elevation in CPK/MB levels compared to the standard value, and alterations in left ventricular ejection fraction (LVEF) assessed through ultrasound, served as indirect indicators. | CEA was selectively decided upon during surgery when total coronary artery occlusion and the impracticality of establishing a successful anastomosis were observed. CEA involved a longitudinal arteriotomy, typically measuring 8–20 mm (closed technique). |
| H.A Huysmans et al., (1988) | saphenous vein, IMA | ECG or enzyme criteria for MI | Since 1977, TEA extended to left coronary artery branches necessitating non-sequential grafting. Since 1978, TEA includes both end-to-side and side-to-side venous grafts, enhancing sequential grafting. |
| Hai Tao Li et al., (2021) | LIMA and saphenous | The 2013 revised universal definition stipulated a diagnosis threshold for myocardial infarction following CABG, set at equal or more than 70 times the 99th percentile of the URL, even in the absence of alterations in ECG when compared to a physiological baseline cardiac troponin level. | In the PDA, closed-traction CEA, following Eryilmaz's technique, removed plaque through gentle proximal and distal traction via the primary incision. The PDA was subsequently connected to the saphenous using 8-0 Suture made from polypropylene. CEA was selectively employed for PDA, a frequently chosen vessel for endarterectomy due to concerns over graft patency. Decisions for CE were guided by preoperative angiography and intraoperative evaluation, conducted only when severe artery occlusion, heavily calcified plaques, and extensive distal stenosis were evident. |
| Hua Yan et al., (2020) (AC group) | NR | Peri-op MI was characterized in alignment with the 4th universal MI definition. | The decision for CE was made during surgery, performed by experienced surgeons using on-pump or off-pump techniques for full revascularization. Closed endarterectomy was favoured in most cases, with patch angioplasty in select patients. |
| Kosaku Nishigawa et al., (2017) | All CEAs required a lengthy arteriotomy and involved the reconstruction of the LAD utilizing the skeletonized ITA. | NR | In cases of severe and circumferential plaques, involvement of side branches, or highly calcified plaques impeding major reconstruction, CEA was performed. CE with stent removal was necessary for cases of extensive in-stent restenosis affecting side branches. The atherosclerotic plaque was meticulously separated from the adventitia. through an extended arteriotomy under direct visualization. To avoid conflicting blood flow through the coronary, the endarterectomy commenced distally from the proximal lesion. The distal incision extended to an unaffected arterial segment, and the endarterectomized left anterior descending was reconstructed using the skeletonized ITA in an onlay-patch configuration. |
| Kosaku Nishigawa et al., (2021) | Saphenous patching with/without utilizing LITA as a graft or overlay patch.  overlay patch grafting utilizing the LITA is favoured due to its superior patency rate, attributed to a larger portion of the reconstructed LAD with healthy LITA intima. This approach consistently involved direct-vision endarterectomy with onlay-patch LITA reconstruction for diffuse LAD disease. | NR | An incision was made in the middle part of the LAD, and the incision's length was determined by the size of the atherosclerotic plaque. The plaque was then separated from the arterial wall using fine forceps and a spatula, particularly when concurrent stent removal was performed. This removal was extended into the branch arteries as far as the spatula could reach, while ensuring the distal branch artery's inner lining was preserved. To avoid affecting the closest narrowing of the artery and to prevent competing blood flow through the natural artery, the proximal end of the plaque was not removed. The far end was precisely cut, and sutures were used to secure the divided inner lining to prevent flap formation. Remaining tissue was subsequently removed, and the interior of the LAD artery was irrigated with a saline solution. Subsequently, the LITA was tailored to match the LAD arteriotomy length and connected to the LAD using sutures. Finally, transit-time flowmetry is employed to assess the graft and anastomosis quality, following conventional criteria. |
| Marco Russo et al., (2016) (DAT group) | Revascularization of the LADA in the entire patient cohort necessitated the utilization of Right or left ITA, and individuals under the age of 60 were assigned RITA grafts for anastomosis with the LADA, whereas the LITA was exclusively reserved for the LCA system. Saphenous vein grafts were employed for other revascularization procedures, with direct visual harvest. | Peri-op MI was characterized by an elevation in postoperative troponin I concentration exceeding 20 ng/ml, concomitant with an elevation in serum creatine kinase-MB levels surpassing the normal range and exceeding 10% of the total creatine kinase levels, along with the onset of abnormalities in electrocardiographic patterns. | Intra-op determination of adjunct CEA was predicated upon the extent of diffuse occlusive pathology within the target coronary vessel. The CEA was carried out by two approaches: the closed method, which entailed making a 10–15 mm longitudinal cut along the coronary artery and careful removal of the sclerotic intimal tissue with fine forceps before anastomosis, with regards the open method, which involved extending the arteriotomy several centimetres in both proximal and distal directions to achieve a more extensive anastomosis. |
| Mohammad Hassan Nemati et al., (2015) | In all patients, the LIMA was utilized for all grafts on LAD, while the saphenous vein was utilized for all other coronaries. | NR | In executing the CEA procedure, the surgical team initiated an incision of approximately 1 to 1.5 cm within the coronary vessels, subsequently conducted a meticulous endarterectomy using a dissector, and proceeded to remove the atheromas from the incision location. |
| Pengxiong Zhu et al., (2019) | LIMA-LAD was used in the 57% of the LAD group, 67% of the LCX group and 68% of the RCA group. | Myocardial infarction was defined as Type 5 MI in accordance with the Third Universal Definition of Myocardial Infarction. | The study used the open CEA for all the patient. |
| Redoy Ranjan et al., (2017) | IMA, saphenous vein, and in some cases radial artery were used for grafting. | NR | The CEAs were executed through a manual, deliberate approach involving the gradual and continuous removal of atheromatous plaque using delicate Ring Forceps. This was followed by the closed techniques and subsequent reconstruction through pre-planned graft anastomosis. |
| Sharaf-Eldin Shehada et al., (2022) (DAT group) | The bypass involved either the use of left IMA in conjunction with a saphenous vein patch or exclusive grafting using the LIMA. | Post-op MI was defined according to the Third Universal Definition of MI. | A modified closed traction CEA technique was used. |
| Simon CY Chow et al., (2021) | Approximately one-third of the grafts utilized were arterial grafts (31.5%), while the majority of grafts consisted of saphenous vein grafts (68.5%). | Periprocedural MI was diagnosed when hemodynamic instability and/or symptoms, along with evidence of new ischemia on ECG or echo, accompanied a substantial increase in troponin levels. Treatment for peri-op MI involved the initiation of triple therapy (heparin, aspirin, and clopidogrel/ticagrelor). Non-procedural MI was defined as MI, either non-ST segment elevation or ST segment elevation, occurring beyond 30 days post-op and characterized by the presence of symptoms, a significant troponin rise, and ECG changes. | The favored approach for CEA in this center was the closed technique. |
| Toshihiro Fukui et al., (2005) | In all cases, the LIMA was utilized as an in-situ on-lay patch graft. | NR | The endarterectomy procedure involved the utilization of an open technique.  The CEA commenced from the distal tip of the stent and expanded in both the upstream and downstream directions. Careful examination of the vessel's outermost layer, where a stent was deployed, was carried out, followed by the removal of the neointima and intima. |
| Yasuyuki Kato et al., (2012) | LITA was employed for grafting to the LAD. | The study characterized peri-op MI as the presence of new Q waves on ECG or a post-op creatine kinase-myocardial band level exceeding 100 IU/L. | In the study, open-CEA technique was employed. The study conducted endarterectomy in cases where extensive calcifications within the LAD would have obstructed LITA anastomosis. In this study dissection of the atheromatous core from the adventitial layer was carried out by using forceps and spatula. The intimal layer of all LAD branches underwent dissection. The separated intimal layer of the distal LAD was secured using 8–0 polypropylene sutures. An on-lay patch anastomosis was fashioned by utilizing a correspondingly sized segment of the LITA, placed over the arteriotomy site. Numerous continuous sutures of 7–0 and 8–0polypropylene thread were employed to reconstruct the LAD, with the graft material comprising the majority of the reconstructed vessel. |
| Z˘ ivojin S. Jonjev et al., (2004) | Typically, LAD or first diagonal branch were anastomosed to skeletonized LITA, however saphenous vein was used for other coronary artery grafts. | The criteria for defining peri-op MI in this study align with those utilized in other pertinent clinical trials such as Minnesota Code and GUARDIAN. | CEA was conducted using a closed and manual surgical approach in all instances. |
| Ziyad M. et al., (2013) | The LIMA was connected to the LAD through an anastomosis. In situations where an extended arteriotomy required repair with a patch, either a segment of the LIMA or a vein was utilized. | NR | The choice to carry out an endarterectomy was determined by reviewing pre-op angiograms and assessing intraoperative observations. This surgical approach was employed to address arteries presenting either complete proximal blockage with no downstream blood flow or partial occlusion with widespread atheroma, where establishing a traditional anastomosis to the target artery was considered unfeasible from a technical standpoint. |

**CABG:** Coronary Artery Bypass Grafting; **CEA:** Coronary endarterectomy; **CK-MB:** Creatine Kinase-MB; **CPK-MB:** Creatine Phosphokinase-MB; **ECG:** Electrocardiogram; **FMJ:** Full Metal Jacket; **IMA:** Internal Mammary Artery; **ITA:** Internal Thoracic Artery; **LAD:** Left Anterior Descending; **LCX:** Left Circumflex; **LITA:** Left Internal Thoracic Artery; **NR:** Not Reported; **PDA:** Posterior Descending Artery; **RCA:** Right Coronary Artery

**Supplementary Table 6 (ST6):** Table listing the study designs of included studies

| Study (leading author) | Study design |
| --- | --- |
| Abdul R Abid et al., (2009) | Prospective cohort |
| Adam El-Gamel et al., (2020) | Retrospective cohort |
| Damien J. Lapar et al., (2011) | Retrospective cohort |
| Dimitar Kyuchukov et al., (2020) | Prospective cohort |
| H.A Huysmans et al., (1988) | Prospective cohort |
| Hai Tao Li et al., (2021) | Prospective cohort |
| Hua Yan et al., (2020) (AC group) | Prospective cohort |
| Kosaku Nishigawa et al., (2017) | Retrospective cohort |
| Kosaku Nishigawa et al., (2021) | Retrospective cohort |
| Marco Russo et al., (2016) (DAT group) | Retrospective cohort |
| Mohammad Hassan Nemati et al., (2015) | Prospective cohort |
| Pengxiong Zhu et al., (2019) | Retrospective cohort |
| Redoy Ranjan et al., (2017) | Retrospective cohort |
| Sharaf-Eldin Shehada et al., (2022) (DAT group) | Retrospective cohort |
| Simon CY Chow et al., (2021) | Retrospective cohort |
| Toshihiro Fukui et al., (2005) | Prospective cohort |
| Yasuyuki Kato et al., (2012) | Retrospective cohort |
| Z˘ ivojin S. Jonjev et al., (2004) | Randomized prospective cohort |
| Ziyad M. et al., (2013) | Retrospective cohort |

|  | Abdul R Abid | Adam El-Gamel | Damien J. Lapar | Dimitar Kyuchukov | H.A Huysmans | Hai Tao Li | Hua Yan | Kosaku Nishigawa 2017 | Kosaku Nishigawa 2021 | Marco Russo | Mohammad Hassan Nemati | Pengxiong Zhu | Redoy Ranjan | Sharaf-Eldin Shehada | Simon CY Chow | Toshihio Fukui | Yasuyuki Kato | Z˘ ivojin S. Jonjev | Ziyad M. |
| --- | --- | --- | --- | --- | --- | --- | --- | --- | --- | --- | --- | --- | --- | --- | --- | --- | --- | --- | --- |
| Cofounding | Moderate | Moderate | Moderate | Moderate | Moderate | Moderate | low | low | low | low | low | low | low | low | low | low | Moderate | low | low |
| Participant Selection | low | low | Moderate | Moderate | high | Moderate | low | low | Moderate | low | low | low | Moderate | low | Moderate | low | Moderate | Moderate | low |
| Classification | low | Moderate | low | low | Moderate | Moderate | low | low | low | low | low | low | low | low | low | low | low | low | low |
| Deviations | low | Moderate | low | low | Moderate | Moderate | low | low | low | low | low | low | low | low | low | low | low | low | low |
| Missing Data | low | low | low | low | low | low | low | low | low | low | low | low | low | low | low | low | low | low | low |
| Measurements | low | Moderate | low | low | Moderate | Moderate | low | low | low | low | low | low | low | low | low | low | low | low | low |
| Reported results Selection | low | Moderate | Moderate | Moderate | Moderate | low | low | low | low | low | low | low | low | low | low | low | low | low | low |
| Overall Result | Moderate | Moderate | Moderate | Moderate | high | Moderate | low | low | Moderate | low | low | low | Moderate | low | Moderate | low | Moderate | Moderate | low |

**Supplementary Table 7 (ST7):** ROBINS-I Assessment for risk of bias.

**Supplementary References:**

26. Kyuchukov D, Zheleva-Kyuchukova I, Nachev G. Antithrombotic regimens in patients after coronary artery bypass grafting and coronary endarterectomy. *Pharmacia*. 2020;67(3):115-120. doi:10.3897/pharmacia.67.e52738
27. Huysmans HA, Aytug Z, Buis B, Verwey H. Treatment of peripheral coronary artery disease by endarterectomy. *Adv Cardiol*. 1988;36:13-18. doi:10.1159/000415609
28. Nishigawa K, Fukui T, Takanashi S. Preoperative SYNTAX score to assess the late outcomes after coronary endarterectomy for the diffusely diseased left anterior descending artery†. *Interact Cardiovasc Thorac Surg*. 2017;25(5):780-784. doi:10.1093/icvts/ivx184
29. Nishigawa K, Fukui T, Takaki J, Takanashi S. Coronary endarterectomy for diffusely diseased coronary artery: An ace in the hole in coronary artery surgery. *JTCVS Tech*. 2021;10:133-137. doi:10.1016/j.xjtc.2021.03.018
30. Nemati MH, Astaneh B, Khosropanah S. Outcome and Graft Patency in Coronary Artery Bypass Grafting with Coronary Endarterectomy. *Korean J Thorac Cardiovasc Surg*. 2015;48(1):13-24. doi:10.5090/kjtcs.2015.48.1.13
31. Ranjan R, Adhikary D, Mandal S, Seedher A, Adhikary AB. Outcome of coronary endarterectomy with coronary artery bypass grafting in patients with diffuse coronary artery disease in Bangladesh: A retrospective cohort study. *JRSM Cardiovasc Dis*. 2017;6:204800401773265. doi:10.1177/2048004017732658
32. Fukui T, Takanashi S, Hosoda Y. Coronary Endarterectomy and Stent Removal in Patients With In-Stent restenosis. *Ann Thorac Surg*. 2005;79(2):558-563. doi:10.1016/j.athoracsur.2004.07.064
33. Kato Y, Shibata T, Takanashi S, Fukui T, Ito A, Shimizu Y. Results of Long Segmental Reconstruction of Left Anterior Descending Artery Using Left Internal Thoracic Artery. *Ann Thorac Surg*. 2012;93(4):1195-1200. doi:10.1016/j.athoracsur.2011.12.059
34. Jonjev ZS, Nićin S, Mujović V, Petrović L, Radovanović N. Prostacyclin Reduces Incidence of Myocardial Damage After Coronary Endarterectomy. *Ann Thorac Surg*. 2004;78(4):1299-1303. doi:10.1016/j.athoracsur.2004.02.132
35. Soylu E, Harling L, Ashrafian H, et al. Does coronary endarterectomy technique affect surgical outcome when combined with coronary artery bypass grafting? Interact Cardiovasc Thorac Surg. 2014;19:848–855
36. Nishi H, Miyamoto S, Takanashi S, et al. Optimal method of coronary endarterectomy for diffusely diseased coronary arteries. Ann Thorac Surg. 2005;79:846–852
37. Tasdemir O, Kiziltepe U, Karagoz HY, et al. Long-term results of reconstructions of the left anterior descending coronary artery in diffuse atherosclerotic lesions. J Thorac Cardiovasc Surg. 1996;112:745–754.
38. Qiu Z, Chen X, Jiang Y, et al. Comparison of off-pump and onpump coronary endarterectomy for patients with diffusely diseased coronary arteries: early and midterm outcome. J Cardiothorac Surg. 2014;9:186
39. Soylu E, Harling L, Ashrafian H, Casula R, Kokotsakis J, Athanasiou T. Adjunct coronary endarterectomy increases myocardial infarction and early mortality after coronary artery bypass grafting: a meta-analysis. Interact Cardiovasc Thorac Surg. 2014 Sep;19(3):462-73. doi: 10.1093/icvts/ivu157. Epub 2014 Jun 3. PMID: 24893867.
40. Balaj I, Jakob H, Haddad A, Mourad F, Haneya A, Ali E, Ryadi N, Thielmann M, Ruhparwar A, Shehada SE. Role of Antiplatelet Therapy in Patients with Severe Coronary Artery Disease Undergoing Coronary Artery Endarterectomy within Coronary Artery Bypass Surgery. J Cardiovasc Dev Dis. 2023 Mar 7;10(3):112. doi: 10.3390/jcdd10030112. PMID: 36975876; PMCID: PMC10051999.
